# Biventricular reverse remodelling achieved through combined surgical and optimal medical therapy in an adolescent female post-Rastelli procedure: a case report

**DOI:** 10.1093/ehjcr/ytaf215

**Published:** 2025-04-28

**Authors:** Natsumi Kashimura, Tomohiro Kaneko, Takao Kato, Sakiko Miyazaki, Tohru Minamino

**Affiliations:** Department of Cardiovascular Biology and Medicine, Juntendo University Graduate School of Medicine, 2-1-1 Hongo, Bunkyo-ku, Tokyo, 113-8421, Japan; Department of Cardiovascular Biology and Medicine, Juntendo University Graduate School of Medicine, 2-1-1 Hongo, Bunkyo-ku, Tokyo, 113-8421, Japan; Department of Cardiovascular Biology and Medicine, Juntendo University Graduate School of Medicine, 2-1-1 Hongo, Bunkyo-ku, Tokyo, 113-8421, Japan; Department of Cardiovascular Biology and Medicine, Juntendo University Graduate School of Medicine, 2-1-1 Hongo, Bunkyo-ku, Tokyo, 113-8421, Japan; Department of Cardiovascular Biology and Medicine, Juntendo University Graduate School of Medicine, 2-1-1 Hongo, Bunkyo-ku, Tokyo, 113-8421, Japan

**Keywords:** Case report, Left ventricular reverse remodelling, Ivabradine, Beta-blocker, Adult congenital heart disease

## Abstract

**Background:**

Advances in medical treatments have allowed many congenital heart disease patients to reach adulthood, resulting in an increase in adult congenital heart disease (ACHD) cases. Managing heart failure (HF) in ACHD patients is a significant concern, particularly due to limited understanding of optimal pharmacological therapies. Conduit dysfunction and right heart failure are common in the late phase after the Rastelli procedure, but right HF is not well understood, and standard medications for acquired HF are not particularly effective. Consequently, there is a knowledge gap regarding optimal treatment strategies.

**Case summary:**

We report the case of a 19-year-old woman with a history of Blalock–Taussig shunt, Rastelli procedure, and reoperation for conduit stenosis. She was diagnosed with chronic active Epstein–Barr virus infection and admitted due to exertional dyspnoea and orthopnoea. On admission, she exhibited signs of acute HF with severe left ventricular dysfunction and conduit stenosis. Initial management with diuretics was insufficient, requiring inotropic support. Right heart catheterization revealed conduit failure, necessitating surgical replacement. Postoperatively, optimized medical therapy, including beta-blockers, ivabradine, vericiguat, and SGLT-2 inhibitors, was administered. Over several months, her biventricular function improved significantly, and she underwent umbilical cord blood transplantation for EBV infection, with a favourable outcome.

**Discussion:**

This case highlights the importance of combining surgical and pharmacological strategies in managing complex ACHD with biventricular dysfunction. Identifying underlying causes, such as chronic myocarditis due to EBV infection, is crucial. Ivabradine allowed for beta-blocker increase, and with newer agents like vericiguat and SGLT-2 inhibitors, significantly improved cardiac function.

**Learning points**

- Identifying and treating the underlying cause of heart failure is crucial for better outcomes in adult congenital heart disease patients.
- Combining surgery and optimized medical therapy can lead to significant improvement in biventricular function in complex congenital heart disease.
- Using ivabradine to manage sinus tachycardia may allow for optimal beta-blocker dosing.

## Introduction

Managing heart failure (HF), the primary cause of mortality in adult congenital heart disease (ACHD) patients, is an immediate concern.^1^ The optimal pharmacological therapy for HF associated with ACHD is not well understood, and only empirical treatment strategies based on those for adult HF patients are recommended.^1,2^ In the late phase after the Rastelli procedure, conduit dysfunction between the right ventricle and the pulmonary artery and the resulting right HF are common issues.^1^ However, right HF is not well understood, and standard medications for acquired HF are not particularly effective.^3–5^ Consequently, there is a knowledge gap regarding optimal treatment strategies.

## Summary figure

**Figure:**
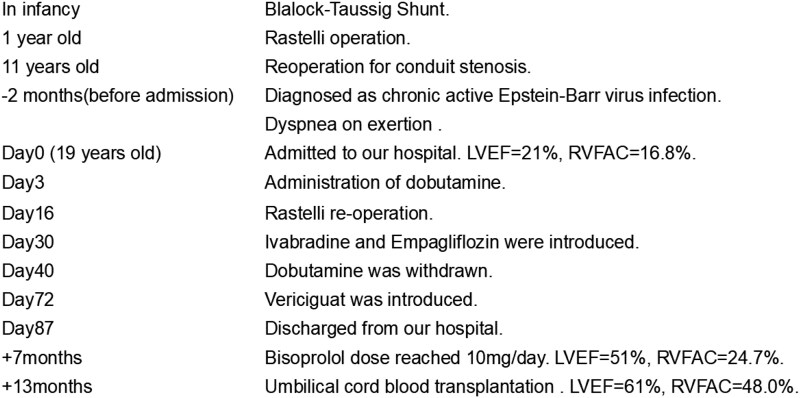


Here, we report a case of acute HF in adolescence, in which biventricular dysfunction and conduit stenosis were observed in the late phase after the Rastelli procedure, and through a combination of surgical treatment and optimal medical therapy, dramatic reverse remodelling of both ventricles was achieved.

## Case presentation

A 19-year-old woman presented to our hospital with complaints of dyspnoea. She was diagnosed with anatomically corrected malposition of the great arteries, coexisting with ventricular septal defect, atrial septal defect, pulmonary valve stenosis, and left pulmonary artery hypoplasia. At birth, the patient exhibited cyanosis, and the SpO₂ in room air was approximately 85%. After she underwent a Blalock–Taussig shunt and Rastelli procedure at the age of 1, the patient’s SpO₂ in room air normalized. A reoperation for conduit stenosis at the age of 11 was performed, and she was followed up at a local medical facility. Three months prior to admission, a transthoracic echocardiography performed for the evaluation of fever of unknown origin revealed a right ventricular fractional area change (RVFAC) of 12%, the peak Rastelli conduit flow velocity was 3.1 m/s, and the left ventricular systolic function was within the normal range. No regurgitation was observed within the Rastelli conduit. Two months prior to admission, she was diagnosed as chronic active Epstein–Barr virus (EBV) infection. Around the same time, she began experiencing exertional dyspnoea, which gradually progressed to orthopnoea at night. Consequently, she was admitted to our hospital.

**Figure 1:**
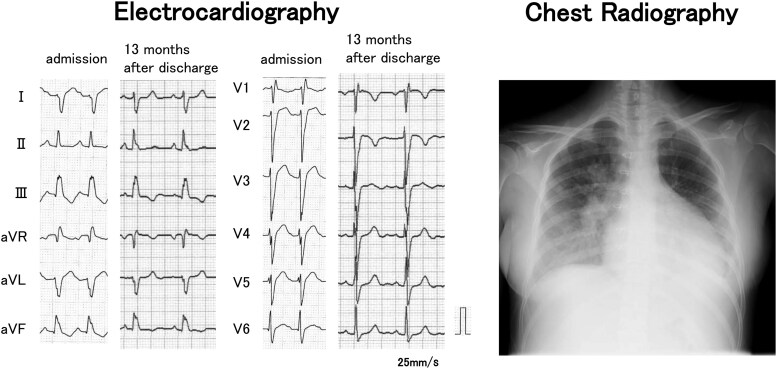
Electrocardiography and chest radiograph at the admission to our hospital. Electrocardiography showed sinus tachycardia and complete right bundle brunch block. Chest radiography revealed cardiomegaly and bilateral pleural effusion. After discharge, the QRS duration improved from 150 ms to 127 ms.

**Figure 2:**
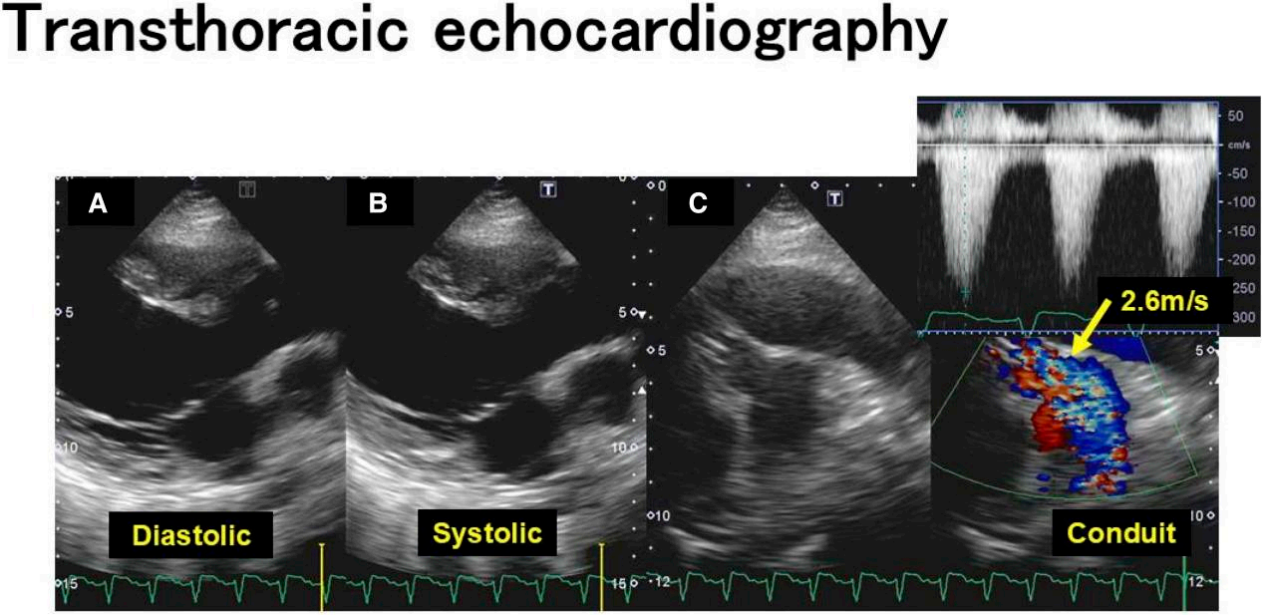
Transthoracic echocardiography at the admission to our hospital. (*A*, *B*) Parasternal long-axis view showing left ventricle at diastolic and systolic. Left ventricular ejection fraction (LVEF) 21%, left ventricular end-diastolic dimension 55 mm, and end-systolic dimension 49 mm. (*C*) Rastelli conduit stenosis which peak flow velocity was 2.6 m/s (indicated by the arrow).

A physical examination revealed blood pressure of 110/82 mmHg, heart rate of 148 beats/min, body temperature of 36.2°C, respiratory rate of 24 breaths/min, and SpO_2_ of 98% (nasal cannula 2 L/min). The jugular veins were distended, and peripheral coldness in the extremities was observed. Electrocardiography showed sinus tachycardia and complete right bundle brunch block. Chest radiography revealed cardiomegaly and bilateral pleural effusion (*Figure 1*). Laboratory data were as follows: serum total bilirubin 40.7 μmol/L (normal range: 6.8–20.5 μmol/L), serum aspartate aminotransferase 296 IU/L (normal range: 5–37 IU/L), serum troponin T 0.049 ng/mL (normal range: ≤ 0.100 ng/mL), N-terminal prohormone of brain natriuretic peptide (NT-proBNP) 11 644 pg/mL (normal range: ≤125 pg/mL). Transthoracic echocardiography was as follows: left ventricular ejection fraction (LVEF) 21%, left ventricular diastolic dimension 55 mm, systolic dimension 49 mm, moderate aortic regurgitation, peak tricuspid regurgitation flow velocity 3.3 m/s, a distended inferior vena cava, RVFAC 16.8%, tricuspid annular plane systolic excursion (TAPSE)/pulmonary artery systolic pressure (PASP) ratio 0.25, and peak Rastelli conduit flow velocity 2.6 m/s (*Figure 2*).

Furosemide and tolvaptan were initiated for acute HF, but on the third day of hospitalization, signs of low cardiac output and compromised perfusion, including hypotension, hyponatremia, and elevated lactate levels, were observed, necessitating the administration of dobutamine. Following the initiation of dobutamine, effective diuresis was achieved, and fluid retention improved. On the eighth hospital day, recurrent low cardiac output and compromised perfusion led to an increase in dobutamine dosage.

To identify the cause of low cardiac output, right heart catheterization and pulmonary artery angiography were performed. Right heart catheterization revealed no elevation in pulmonary artery wedge pressure, but a stenosis between the distal conduit and right ventricle with a pressure gradient of 32 mmHg was observed. Pulmonary angiography revealed hypoplasia of the left pulmonary artery and moderate regurgitation within the conduit. We determined that the primary contributor to the low cardiac output was conduit failure and chose to proceed with surgical replacement of the conduit to relieve the stenosis. The conduit was compressed by a thick coating of Gore-Tex sheet. The inner surface of the removed conduit was covered with vegetation-like material. Postoperatively, hemodynamics promptly improved, allowing for the discontinuation of inotropic agents. On the other hand, biventricular dysfunction persisted, with an LVEF of 22% and an RVFAC of 24.2% (see Supplementary material online, *Video S1*). The right ventricular to pulmonary arterial coupling improved (TAPSE/PASP ratio increased from 0.25 to 0.54).

Differential diagnoses for left ventricular dysfunction (LVD) included cardiomyopathy, myocarditis, tachycardia-induced cardiomyopathy, and LVD due to ventricular–ventricular interaction and electrical dyssynchrony. To address this concern, preoperative cardiac MRI and myocardial biopsy were performed. The results of the cardiac MRI were as follows: LVEF 16.6%, left ventricular end-diastolic volume 111.5 mL (75.9 mL/m^2^), left ventricular end-systolic volume 93.0 mL (63.3 mL/m^2^), left ventricular mass 81.8 g (left ventricular mass/volume ratio 0.73), right ventricular ejection fraction 22.7%, right ventricular end-diastolic volume 147.8 mL (100.5 mL/m^2^), and right ventricular end-systolic volume 114.3 mL (77.8 mL/m^2^), right ventricular mass 26.9 g (right ventricular mass/volume ratio 0.19). Late gadolinium enhancement (LGE), likely reflecting postoperative changes, was observed in the subepicardial basal lateral wall. The delayed enhancement images from cardiac MRI and the results of myocardial biopsy suggested that chronic myocarditis associated with chronic active EBV infection was the cause of LVD.

**Figure 3:**
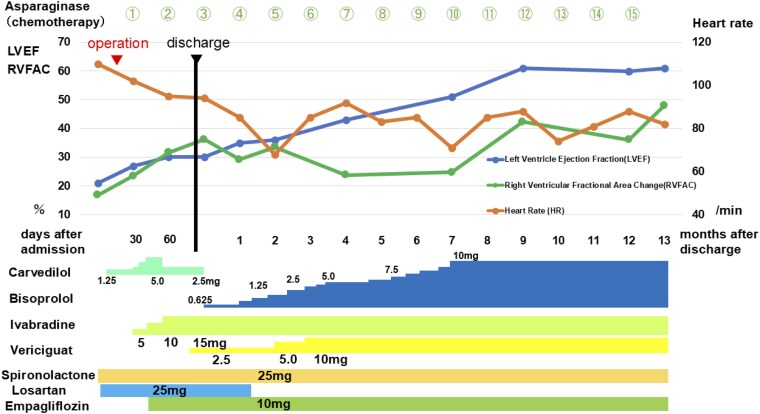
Clinical course. LVEF, left ventricular ejection fraction; RVFAC, right ventricular fractional area change.

Therefore, a treatment plan was instituted to promote left ventricular reverse remodelling with optimal medical therapy, similar to that used in adults, and chemotherapy (l-asparaginase) for chronic active EBV infection. Specifically, in addition to mineralocorticoid receptor antagonist (spironolactone) and the initiation of the angiotensin II receptor blocker (ARB) (losartan), a gradual escalation of carvedilol over a period of 2 weeks was implemented (*Figure 3*). Due to a decrease in blood pressure, we were compelled to reduce the dose of carvedilol. Additionally, since the patient had persistent sinus tachycardia from the preoperative period, we initiated the If-channel inhibitor (ivabradine) and gradually increased its dose over 1 month (*Figure 3*). Following the initiation of ivabradine, there was a clear improvement in tachycardia, an increase in blood pressure, and overall stabilization. Soluble guanylate cyclase receptor stimulator (vericiguat) and sodium-glucose co-transporter 2 (SGLT-2) inhibitors (empagliflozin) were also started simultaneously. The patient was discharged on the 87th hospital day. At the time of discharge, the NT-proBNP level improved from 4449 pg/mL just before surgery to 1478 pg/mL. During outpatient treatment, it was necessary to increase the dosage of the beta-blocker to lower the heart rate. Due to dizziness caused by hypotension, carvedilol was switched to bisoprolol and losartan was discontinued, resulting in symptom improvement. Bisoprolol could then be titrated up to 10 mg per day. As a result of the stepwise adjustment of medications over the 7-month period following discharge (*Figure 3*), biventricular systolic function improved, reaching normal levels by 7 months post-discharge (see Supplementary material online, *Video S1*). The QRS duration improved from 150 ms to 127 ms (*Figure 1*). The standard deviation of time to onset or peak longitudinal strain in 6 right ventricular segments^6^ improved from 68.9 ms to 56.8 ms, and that of time to onset or peak circumferential strain in 6 segments on the left ventricular short-axis plane^7^ improved from 97.3 ms to 27.8 ms. The NT-proBNP level improved to 435 pg/mL. Although the EBV-DNA level at 13 months post-discharge was 7.03 Log IU/mL (normal range: <the limit of detection), showing no improvement compared with the pre-chemotherapy level of 6.76 Log IU/mL, serum troponin T levels improved to 0.006 ng/mL, and the patient subsequently underwent umbilical cord blood transplantation. Post-transplantation, persistent fever ceased, and the patient’s condition has been excellent without readmission for HF for 1 year (*Figure 3*).

## Discussion

We present a case in which a combination of surgical treatment and thorough optimization of HF medications, following the same protocols as those for adults, resulted in dramatic biventricular reverse remodelling.

In this case, moderate or greater conduit stenosis and regurgitation within the conduit may have led to right ventricular dysfunction, subsequently causing secondary LVD through ventricular–ventricular interaction. Tachycardia and electrical dyssynchrony associated with conduction disturbances may also have contributed to LVD. In tetralogy of Fallot, which shares similar haemodynamics, LVD is known to be caused by aortic valve insufficiency, ventricular–ventricular interaction, and electrical dyssynchrony.^1^ Surgical intervention for conduit stenosis and dysfunction may have contributed to the improvement of LVD through the improvement of right ventricular dysfunction, mediated by ventricular–ventricular interaction. In patients with repaired tetralogy of Fallot, LVD is correlated with right ventricular dysfunction, and while 20.9% of patients develop LVD, severe dysfunction is extremely rare, occurring in only 0%–1% of cases.^8,9^ The patient presented with severe LVD, and the results of cardiac MRI and myocardial biopsy suggest that myocarditis associated with chronic active EBV infection might have also influenced the LVD. Although contrast-enhanced cardiac MRI has been shown to be useful in detecting acute myocarditis, LGE is primarily associated with myocardial necrosis and interstitial oedema, which characterize the inflammatory process in the acute phase. Notably, 32% of cases of chronic myocarditis diagnosed by endomyocardial biopsy do not exhibit LGE.^10^ In this case, endomyocardial biopsy confirmed myocarditis related to chronic active EBV infection, and chemotherapy with L-asparaginase was administered as a bridge to umbilical cord blood transplantation. As we previously reported,^11^ including this case, L-asparaginase is effective for chronic active EBV infection, and chemotherapy might have contributed to the improvement of cardiac function. In this case, although EBV-DNA levels did not improve prior to umbilical cord blood transplantation, serum troponin T levels decreased. It has been reported that serum troponin T levels are elevated in chronic myocarditis.^12^ Due to limited literature regarding myocarditis associated with chronic active EBV infection and optimal methods for the disease monitoring, further research is necessary to clarify the impact of chemotherapy on cardiac function.

This case highlights the importance of optimizing HF medications, including novel agents. In this patient with sinus tachycardia, increasing the dose of beta-blockers was challenging due to hypotension, so ivabradine was prescribed. Beta-blockers are important treatments for improving left ventricular remodelling and the prognosis of HF in ACHD patients, but their effectiveness for right HF is limited.^3,13,14^ Similarly, the efficacy of renin-angiotensin-aldosterone system inhibitors in ACHD is also restricted.^4,5^ The ARBs are associated with improvements in right ventricular ejection fraction in subgroups with non-restrictive right ventricular physiology and incomplete remodelling (QRS fragmentation and a history of pulmonary valve replacement),^4^ while the angiotensin-converting-enzyme inhibitors increase LVEF in subgroups of patients with restrictive right ventricular physiology.^5^ Ivabradine lowers heart rate by inhibiting the If-channel without exerting negative inotropic effects.^15^ The reduction in heart rate promotes decreased myocardial oxygen consumption, prolonged diastolic time, and increased coronary blood flow, thereby improving cardiac output and LVEF.^16–18^ In this case of biventricular dysfunction, although ARB had to be discontinued due to hypotension, the combination of ivabradine and a beta-blocker may have played a significant role in achieving adequate heart rate reduction. In this case, not only was there a reduction in heart rate, but the shortening of the QRS duration may have also contributed to the improvement in LVEF. It has been suggested that improvement in ventricular synchronization is strongly associated with favourable cardiac reverse remodelling.^19^ Takenaka *et al*.^20^ have reported in a case study that SGLT-2 inhibitors may promote electrical and mechanical reverse remodelling, suggesting that SGLT-2 inhibitors may have contributed to the improvement of conduction disturbances in this case. In this case, vericiguat may also have contributed to the improvement of haemodynamics. Vericiguat has been reported to promote left ventricular reverse remodelling and improve right ventricle-pulmonary artery coupling, thereby enhancing the prognosis of adult patients with HF with reduced LVEF.^21,22^ Reports on their efficacy in patients with right HF and ACHD are limited, and further investigation is needed.^21,23^ Recurrent HF has been reported in patients with improved left ventricular function who discontinue pharmacological therapy,^24^ indicating that these treatments need to be continued in the future.

## Conclusion

In conclusion, in cases of ACHD with concomitant LVD, it is crucial to identify the underlying cause, address the causative disease, and optimize pharmacological therapy for HF. This case highlights the potential effectiveness of pharmacotherapy for HF associated with ACHD and serves as a stimulus for further research in the future.

## Supplementary Material

ytaf215_Supplementary_Data

## Data Availability

The data underlying this article cannot be shared publicly due to the privacy of individuals that participated in the study. The data will be shared on reasonable request to the corresponding author.

## Lead author biography

She graduated from Tokyo Women’s Medical University in Tokyo, Japan and then worked as a junior resident at Juntendo University Nerima Hospital. After that, she served as a resident in the Department of Cardiovascular Biology and Medicine at Juntendo University.
